# New insights into negative effects of lithium on sea urchin *Paracentrotus lividus* embryos

**DOI:** 10.1038/srep32157

**Published:** 2016-08-26

**Authors:** Nadia Ruocco, Maria Costantini, Luigia Santella

**Affiliations:** 1 Department of Biology and Evolution of Marine Organisms, Stazione Zoologica Anton Dohrn, Villa Comunale, 80121 Napoli, Italy; 2 Department of Biology, University of Naples Federico II, Complesso Universitario di Monte Sant’Angelo, Via Cinthia, 80126, Napoli, Italy; 3 Bio-Organic Chemistry Unit, Institute of Biomolecular Chemistry-CNR, Via Campi Flegrei 34, Pozzuoli, Naples 80078, Italy

## Abstract

The diffuse use of lithium in a number of industrial processes has produced a significant contamination of groundwater and surface water with it. The increased use of lithium has generated only scarce studies on its concentrations in ambient waters and on its effects on aquatic organisms. Only few contributions have focused on the toxicity of lithium in marine organisms (such as marine animals, algae and vegetables), showing that the toxic effect depends on the animal species. In the present study we describe the morphological and the molecular effects of lithium chloride (LiCl), using the sea urchin *Paracentrotus lividus* as a model organism. We show that LiCl, if added to the eggs before fertilization, induces malformations in the embryos in a dose-dependent manner. We have also followed by RT qPCR the expression levels of thirty seven genes (belonging to different classes of functional processes, such as stress, development, differentiation, skeletogenesis and detoxifications) to identify the molecular targets of LiCl. This study opens new perspectives for the understanding of the mechanism of action of lithium on marine organisms. The findings may also have relevance outside the world of marine organisms since lithium is widely prescribed for the treatment of human bipolar disorders.

Lithium is abundantly present in the earth’s crust^1^: it typically forms salts like phosphates, silicates, and micas^2^,^3^. Mobilized by weathering processes, lithium is transported into soils where it can be potentially toxic to plants^3^. It is present in river’s water, in brine, and in drinking waters. The lithium content ingested from food has been estimated at 2 milligrams per day, the primary source being grains and vegetables^3^.

The industrial use of lithium, e.g., for the production of metal alloys, ceramics, TV screens, color films, pool cleaning chemicals, contributes to environmental pollution^4^. Lithium is also involved in the production of synthetic rubber, pharmaceuticals, lubricants, coolant in nuclear reactors and batteries^5^,^6^,^7^. A source on environment contamination by lithium is the widespread habit of disposing spent batteries along with normal garbage^6^.

Only scarce information is available on the inhalation toxicity of lithium, although nasal irritations and coughing were reported in occupationally exposed workers^4^. Some data are available on rats exposed for 4 h to aerosols containing 80% lithium carbonate: they displayed ulcerative rhinitis often accompanied by squamous metaplasia, necrotic laryngitis and respiratory difficulties^8^. Moreover, signs of anorexia and dehydration were observed, showing that inhaled lithium crosses the lipid-rich layer lining the lung alveolar surface to reach the kidney via the blood^9^. Lithium can accumulate in marine animals, algae, vegetables. It is important to consider that grains and vegetables are the primary dietary sources of lithium, contributing from 66% to 90% of the total lithium intake^3^. To date, clear results on the concentration of lithium in marine environment are not available to evaluate its toxicity. A few data are available on the distribution and toxicity of lithium in the aquatic environment in the United States^10^.

Lithium toxicity has raised concerns, given its widespread use to treat bipolar disorders^11^,^12^. Doses resulting in serum levels from 0.8 to 1.2 mM are recommended and widely used, but they are associated with a higher incidence of side effects such as gastro intestinal, renal, neurological and endocrine disorders^13^,^14^,^15^,^16^,^17^,^18^. Despite its theraupeutic use, little is known about the way lithium can alter neurotransmission. Lithium has been shown to decrease the level of neuronal inositol through the inhibition of inositol monophosphatase (IMPase), which converts myo-inositol monophosphates to myo-inositol, to reconstitute the membrane phospholipids, phosphatidylinositol 4,5-bisphosphate (PIP_2_) pool. Eventually, the latter generates the Ca^2+^-mobilizing second messenger D-myo-Inositol-1,4,5-trisphosphate (InsP_3_) and diacylglycerol (DAG). The lithium-induced inositol depletion, and the consequent disturbance of the Ca^2+^ signaling operation, affect the behavior of neurons in culture, impairing neurotransmission and altering growth cone and the cytoskeleton^19^,^20^.

A teratogenic effect of lithium on the development on *Dictyostelium*, zebrafish, *Xenopus* and sea urchin organisms has also been documented^21^. It has been shown that lithium perturbs pattern formation resulting in embryos that have a deformed dorso-ventral axis^22^,^23^,^24^,^25^,^26^. In line with a role of lithium in reducing cellular levels of myo-inositol and PIP_2_ concentrations, myo-inositol can protect *Xenopus* and sea urchin embryos by counteracting the teratogenic effect^27^. The administration of lithium, or of another IMPase inhibitor (L690,330), after fertilization of sea urchin, has been shown to block the cell cycle transitions in the first cleavage of embryos, and to induce profound effects on sea urchin development^28^. These effects were reversed by myo-inositol^29^,^30^. However, it was also shown that the complete inhibition of IMPase had no effect on the morphogenesis of *Xenopus* embryos, thus, a new hypothesis for the molecular mechanism of lithium on development was proposed. I.e., it was claimed that lithium inhibits glycogen synthase kinase- kinase-3 (GSK-3), which regulates cell lineage determination in several embryos^31^. The issue is controversial, as more recent results of *in vivo* studies have revealed that lithium is not a specific, nor a potent inhibitors of GSK3^32^.

Various studies suggest that some harmful effects of lithium could be related to oxidative stress^33^,^34^, whereas at therapeutic concentration lithium was found to confer protection against toxic stimuli inducing oxidative stress and apoptosis^35^,^36^,^37^,^38^. At the moment, the fine details of the pharmacological and toxicological mechanisms of the effects of lithium remain poorly understood^39^,^40^.

The aim of the present study was to explore the effects of lithium using *Paracentrotus lividus* embryos as model organisms. The sea urchin *P. lividus*, is a good model system to study the ecotoxicological response of marine invertebrates to environmental pollutants for several reasons: its ecological relevance, benthic and relatively sedimentary lifestyle, rapid response and high sensitivity to many types of contaminants, its transparent embryos that grow rapidly in the laboratory and its long reproductive period. We thus decided to adopt it for our study, and treated embryos with increasing concentrations of lithium chloride to analyze the ensuing morphological changes and to define the mechanism of their production. We also monitored the response to the lithium exposure of thirty seven genes that have key roles in a broad range of functional responses, such as development, differentiation and detoxification processes.

## Methods

### Ethics Statement

*Paracentrotus lividus* (Lamarck) sea urchins were collected from a location that is not privately-owned or protected in any way, according to Italian legislation of the Marina Mercantile (Decreto del Presidente dellaRepubblica DPR 1639/68, 09/19/1980 confirmed on 01/10/2000). The field studies did not involve endangered or protected species. All animal procedures were in compliance with the guidelines of the European Union (Directive 609/86).

### Gamete collection, embryo culture, exposure to LiCl and morphological analysis

Adult sea urchins of the species, *P. lividus*, were collected during the breeding season by scuba-diving in the Gulf of Naples, transported in an insulated box to the laboratory within 1 hour after collection and maintained in tanks with circulating sea water until testing. Sea urchins were injected with 0.5 M KCl through the peribuccal membrane to obtain the emission of gametes. Eggs were washed with filtered sea water (FSW) and kept in FSW until use. Concentrated ‘dry’ sperm was collected and kept undiluted at +4 °C until use.

Lithium chloride was added to the eggs 10 minutes before fertilization (mpf) and 10 minutes post-fertilization (mpf) at the following concentrations: 1, 2, 3, 4, 5, 10 and 80 mM. Eggs were fertilized utilising sperm-to-egg ratios of 100:1 for both controls and treated embryos. Fertilized eggs were kept at 20 °C in a controlled temperature chamber on a 12 h:12 h light:dark cycle. Controls were also performed in FSW without LiCl.

After 48 hour of incubation and at 1 week post-fertilization, morphological malformations were determined for at least 200 plutei from each female (fixed in formaldehyde 4% in FSW) using a light microscope (Zeiss Axiovert 135TV, Carl Zeiss, Jena, Germany), in comparison to control embryos in FSW without LiCl.

For recovery experiments the procedure for the treatments with the LiCl was the same as reported above, adding LiCl before fertilization. The experiments have been performed at the concentration of 80 mM and embryos have been washed twice at different development: 10, 20 and 40 minutes post-fertilization (mpf). Embryos were grown at the pluteus stage. Controls were also performed, incubating embryos with LiCl, without washing. The number of abnormal embryos was evaluated by fixing embryos in formaldehyde (4% in FSW) and counting under the light microscope.

In the case of experiments to determine the teratogenic concentration for the molecular analysis, eggs were treated with LiCl at specific concentrations included in the range between 1.5–2 mM. Abnormal plutei were counted under the light microscope.

All experiments were conducted in triplicate using three egg groups collected from three different females.

Statistical analysis was performed using GraphPad Prism version 4.00 for Windows (GraphPad Software, San Diego California USA).

### RNA extraction and cDNA synthesis

About 8000 eggs in 50 mL of FSW were treated with 2 mM of LiCl for 10 minutes and then fertilized. Samples were then collected at 5, 21 and 48 hours post-fertilization (hpf) by centrifugation at 1800 relative centrifugal force for 10 minutes in a swing out rotor at 4 °C. The pellet was washed with phosphate buffered saline and then frozen in liquid nitrogen and kept at −80 °C. Experiments were conducted in triplicate using three egg groups collected from three different females.

Total RNA was extracted using GenElute^TM^ Mammalian Total RNA Miniprep Kit (SIGMA-ALDRICH^®^) according to the manufacter’s instructions. Contaminating DNA was degraded by treating each sample with a DNaseRNase-free kit (Roche, Milan, Italy) according to the manufacturer’s instructions. The amount of total RNA extracted was estimated by the absorbance at 260 nm and the purity by 260/280 and 260/230 nm ratios, using a NanoDrop spectrophotometer (ND-1000 UV-Vis Spectrophotometer; NanoDrop Technologies, Wilmington, DE, USA). The integrity of RNA was evaluated by agarose gel electrophoresis. Intact rRNA subunits (28S and 18S) were observed on the gel indicating minimal degradation of the RNA. For each sample, 600 ng of total RNA extracted was retrotranscribed with an iScript™ cDNA Synthesis kit (Bio-Rad, Milan, Italy), following the manufacturer’s instructions. Synthetized cDNA was used in Real-Time qPCR experiments without dilution.

### Gene expression by Real-Time qPCR

For all real-time qPCR experiments, the data from each cDNA sample were normalized using the zinc-finger transcription factor *Pl-Z12–1* mRNA as endogenous control, because its expression is relatively constant in all developmental stages examined^41^. The expression level of thirty seven genes (see Table S1)^42^ were followed by Real Time qPCR.

Diluted cDNA was used as a template in a reaction containing a final concentration of 0.3 mM for each primer and 1 × FastStart SYBR Green master mix (total volume of 10 μL) (Applied Biosystems, Monza, Italy). PCR amplifications were performed in a ViiATM7 Real Time PCR System (Applied Biosystems, Monza, Italy) thermal cycler using the following thermal profile: 95 °C for 10 min, one cycle for cDNA denaturation; 95 °C for 15 s and 60 °C for 1 min, 40 cycles for amplification; 72 °C for 5 min, one cycle for final elongation; one cycle for melting curve analysis (from 60 °C to 95 °C) to verify the presence of a single product. Each assay included a no-template control for each primer pair. To capture intra-assay variability, all real-time qPCR reactions were carried out in triplicate. Fluorescence was measured using ViiATM7 software (Applied Biosystems, Monza, Italy). The expression of each gene was analysed and internally normalized against *Pl-Z12-1* using REST software (Relative Expression Software Tool, Weihenstephan, Germany) based on the Pfaffl method^43^,^44^. Relative expression ratios above one cycles were considered significant. Experiments were repeated at least twice. Experiments were conducted in triplicate using three egg groups collected from three different females. Statistical analysis was performed using GraphPad Prism version 4.00 for Windows (GraphPad Software, San Diego California USA).

## Results

### Effects of LiCl on sea urchin development

**Figure 1:**
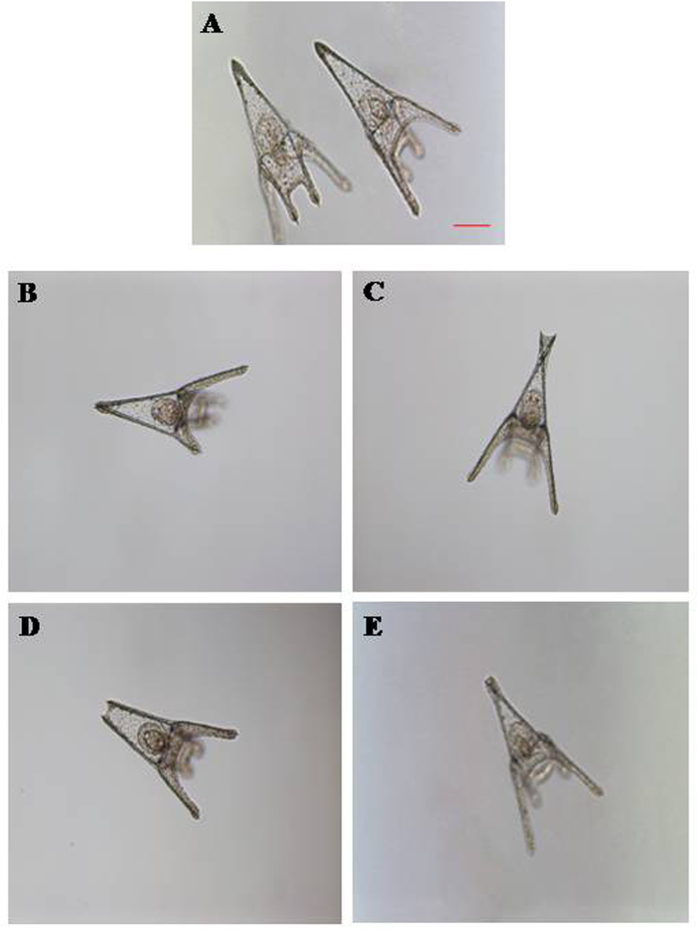
Malformations induced by LiCl. Examples of malformations induced in (**B**–**E**) *Paracentrotus lividus* plutei treated with LiCl from 1.0 to 10 mM and observed at 48 hpf in comparison with (**A**) embryos in sea water without LiCl. Photos were taken with a Zeiss Axiovert 135TV, 10x/0.30, magnification / numerical aperture. Bar, 50 μM.

Considering that 1 mM is the optimal range concentration of lithium plasma levels that is maximally beneficial for the treatment of bipolar disorder, we used this concentration as a starting point to study the effects of lithium on sea urchin development. In fact, increasing concentrations (1, 2, 3, 4, 5 and 10 mM) of LiCl were added separately to *P. lividus* eggs at 10 mbf and 10 mpf. Morphological analysis at the pluteus stage (48 hpf) revealed that the treatment at different concentrations tested induced the same malformations, which principally affected the arms, spicules and apex, in comparison with control embryos in FSW without LiCl (Fig. 1A). Such plutei had not-well-formed arms (Fig. 1B), or a poorly-formed apex with the spicules that appeared crossed at the apex (Fig. 1C) or disjoined at the tip (Fig. 1C), or arms that appeared broader (Fig. 1D) than in the controls (Fig. 1A).

**Figure 2:**
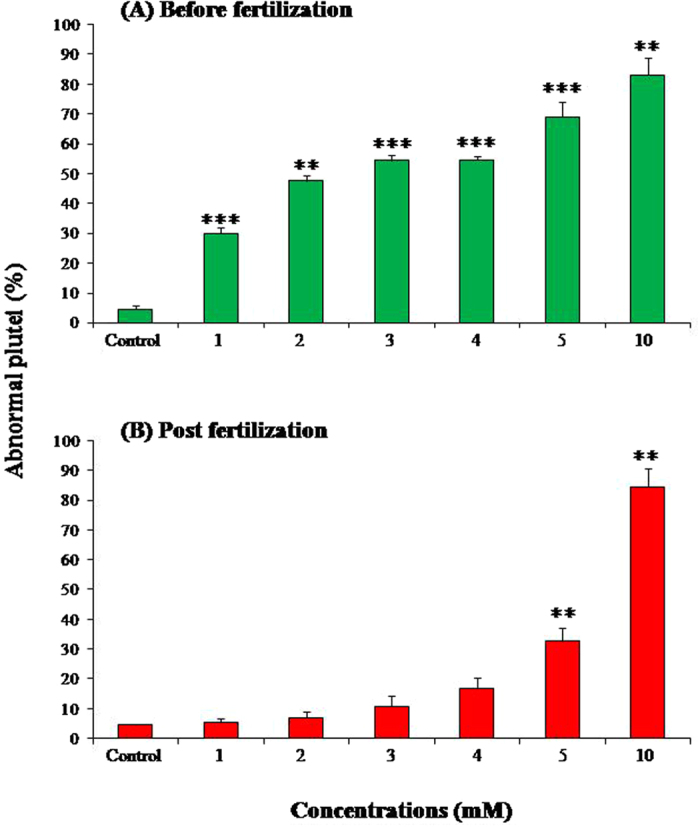
Percentage of abnormal plutei. Percentage of abnormal plutei (at 48 hpf) in *P. lividus* eggs incubated with LiCl 10 minutes before fertilization and then fertilized (**A**) and in eggs treated with LiCl 10 minutes after fertilization (**B**). Different concentrations of LiCl were used: 1, 2, 3, 4, 5 and 10 mM. Significant differences compared to the control (4.3 ± 0.8 abnormal embryos): ***p < 0.001 (Student’s t-test, GraphPad Software Inc., San Diego, CA, USA). Experiments were conducted in triplicate using three egg groups collected from three different females.

**Figure 3:**
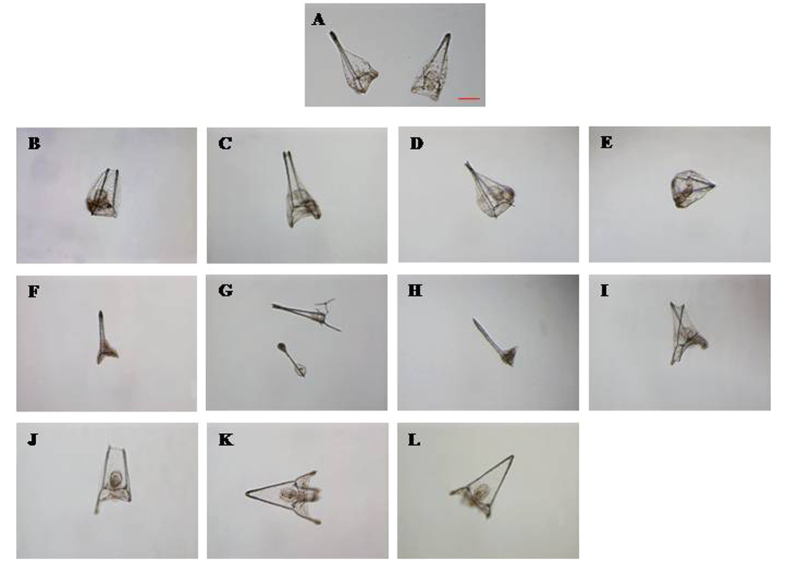
Malformations in sea urchin embryos at one week after fertilization. Examples of malformed embryos induced in *P. lividus* embryos at one week of incubation with LiCl 5 and 10 mM (**B**–**L**), in comparison with control embryos in sea water without LiCl (**A**). Photos were taken with a Zeiss Axiovert 135TV, 10x/0.30 (magnification/numerical aperture). Bar, 50 μM.

**Figure 4:**
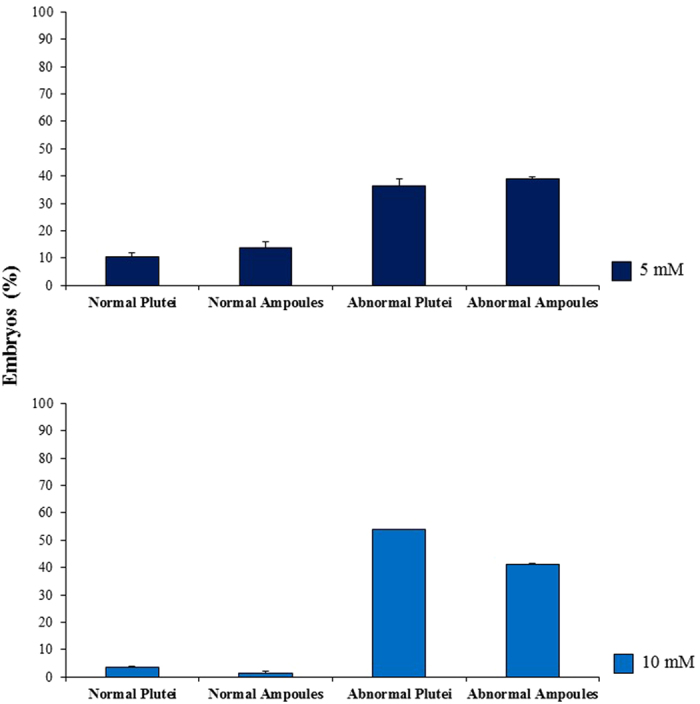
Percentage of malformed embryos at one week after fertilization. Percentage of normal ampoules, normal plutei, abnormal ampoules and abnormal plutei in samples incubated with LiCl 5 and 10 mM at one week after fertilization. Values in graphs represent the mean ± SD (N = 3). Significant differences with the control (embryos grown in FSW): ***p < 0.001.

Our results showed that the effects of LiCl were stronger before fertilization. At the concentration of 1 mM LiCl we observed an increase of about 30% malformed plutei in comparison with control embryos developed in FSW without LiCl. At 10 mbf a significant increasing percentage of abnormal plutei was observed from 1 mM up to 10 mM (Fig. 2). On the other hand, when LiCl was added after fertilization, a significant increase in the percentage of abnormal plutei was only detected at 5 and 10 mM. The times of exposure to 5 and 10 mM LiCl (added before fertilization) were extended to one week post-fertilization (wpf) to follow the fate of the plutei. After the pluteus stage, embryos began the retraction of the arms, firstly assuming a pyramid shape and then a characteristic “ampoule-like” shape (Fig. 3A)^45^. The treatment with LiCl (5 and 10 mM) induced malformations of the embryos at one wpf as reported in Fig. 3. In fact, microscopic observations showed that some embryos assumed the ampoule shape but the spicules appeared not-joined (Fig. 3B,C) or crossed at the tip (Fig. 3D). In these embryos the general body plan was definitely compromised and the entire body of the embryos was malformed (Fig. 3E–I). Several embryos had the pyramid shape and poorly-retracted and degraded arms (Fig. 3J–L). On the basis of these observations, the percentages of normal and abnormal embryos in samples treated with 5 and 10 mM LiCl and in the control were calculated after one week post-fertilization (Fig. 4): the percentage of abnormal plutei and ampoules increased at the concentration of 10 mM.

**Figure 5:**
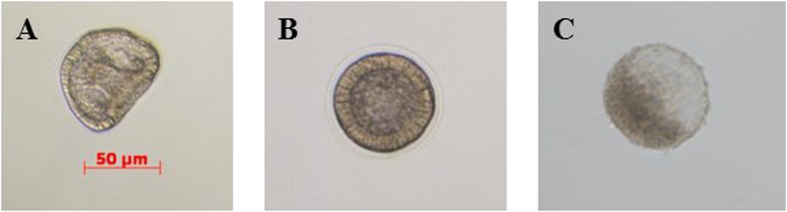
Experiments with LiCl 80 mM. Embryos observed at 21 hpf (**B**) and 48 hpf (**C**), when eggs were incubated with LiCl 80 mM and then fertilized; (**A**) shows control embryos (embryos in sea water without LiCl) at 21 hpf.

**Figure 6:**
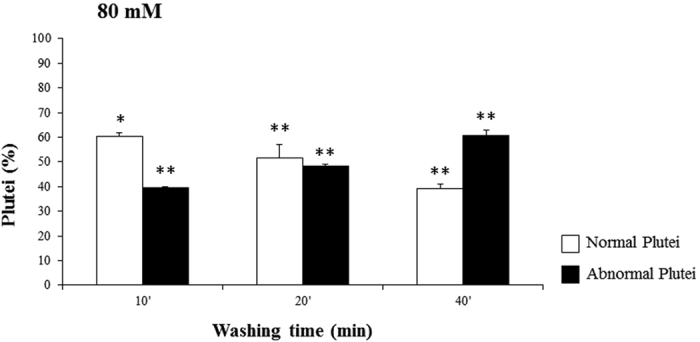
Recovery experiments with LiCl 80 mM. Percentage of normal and abnormal *P. lividus* embryos after exposure to LiCl 80 mM. Eggs were incubated for 10 minutes with LiCl, then fertilized and washed at different times after fertilization, 10, 20 and 40 mpf. Significant differences with the control (not washed embryos): *p < 0.05, **p < 0.01, ***p < 0.001. One-way ANOVA (p < 0.05), with Tukey’s Multiple Comparison Test.

We also performed experiments adding LiCl at the concentration of 80 mM before fertilization. At this concentration LiCl blocked embryonic development at the blastula stage before the hatching (Fig. 5). Observations of embryos in 80 mM lithium after 48 hpf revealed that the blocked embryos then died. Recovery experiments were also performed to establish if sea urchin embryos exposed to 80 mM lithium were able to recover. Eggs were incubated with LiCl, fertilized, and washed at three different times after fertilization: 10 mpf, 20 mpf and 40 mpf. After washing, embryos were grown to the pluteus stage to calculate the number of abnormal embryos. The results indicated that the embryos were able to recover. In fact, when washed, they were able to grow up to the pluteus stage (Fig. 6), even if some of them were malformed. In particular, we detected an increasing percentage of abnormal plutei with the increasing of the washing times. These results suggested that the time in which the embryos remained in contact with the litium was important: i.e., the number of malformed embryos increased with the time of exposure to lithium. The development of these embryos was followed until one week after the washing, showing that the percentage of abnormal embryos remained more or less the same (Supplementary Figure S1).

### Effects of LiCl on gene expression

Embryos were treated with 2 mM LiCl, producing about 45% abnormal embryos (see Fig. 2). Samples were collected at different development times after fertilization, corresponding to the stages of early blastula (5 hpf), late gastrula (21 hpf) and pluteus (48 hpf). To detect potential gene targets of LiCl, the expression levels of thirty seven genes were followed by Real Time qPCR. These genes belong to different functional classes: stress-related genes, genes involved in development and differentiation processes, genes involved in detoxification and skeletogenesis processes (Supplementary Figure S2). The control gene for Real Time *q*PCR was *Pl-Z12-1*; variation of expression levels were calculated as relative expression ratios of the analyzed genes with respect to control embryos in sea water without LiCl. Only expression level greater than one-fold with respect to the controls were considered significant.

**Figure 7:**
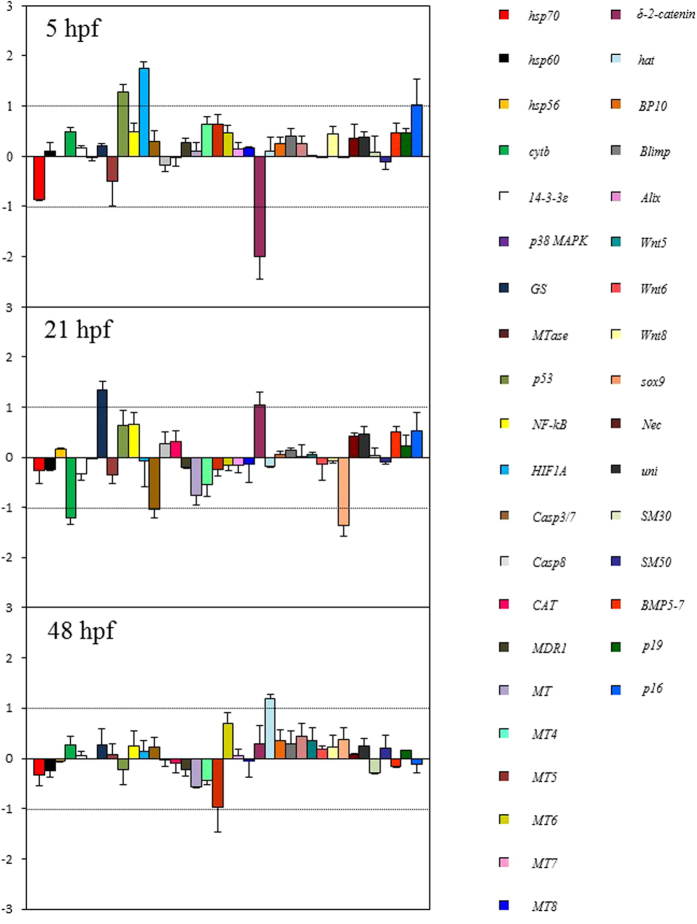
Gene expression by Real Time qPCR. Histograms showing the differences in expression levels of the genes analysed by Real Time qPCR. *P. lividus* embryos were grown in the presence of LiCl and collected at different developmental stages: early blastula (5 hpf), late gastrula (21 hpf) and pluteus (48 hpf). Data are reported as a fold difference compared to control embryos in sea water without HEPES (mean ± SD). Fold differences greater than ±1 (see dotted horizontal guidelines at values of 1 and −1) were considered significant.

At early blastula stage (5hpf) three genes, *HIF1A*, *p53* and *p16* resulted up-regulated with an increase of 1.2-, 1.7-, 1-fold, respectively; *δ-2-catenin* was down-regulated with a 1.9-fold decrease in expression levels (Fig. 7). At the late gastrula stage, the *GS* and *δ-2-catenin* genes showed an increase of expression levels (1.3- and 1-fold, respectively), whereas two other genes were negatively affected: *cytb* (down-regulated by 1.2-fold) and *sox-9* (down-regulated by 1.3-fold).

At the pluteus stage (48 hpf), only the up-regulation of the developmental gene *hat* was detected, with an increase of 1.1-fold with respect to the control.

## Discussion

The toxic effect of LiCl on embryonic development has been explored on different model organisms, such as squid, *Xenopus*, zebrafish and sea urchin^46^,^47^,^48^,^49^,^50^. The results have shown that LiCl inhibits the development along the animal vegetal axis and anterior midline of the squid embryos^51^. In *Xenopus laevis* it alters the axial patterning of the body, producing embryos with reduced posterior but exaggerated anterior structures and embryos with truncation of anterior structures^52^,^53^. LiCl also induces significant phenotypic abnormalities in zebrafish development, such as pericardial and yolk sac oedema, dispersed pigment cells^54^.

Work on *P. lividus* sea urchin embryos, the species used in the present study, has demonstrated that lithium acts as a larval vegetalizing agent, i.e., it acts by enhancing the endoderm-mesoderm structures at the expense of the ectoderm^55^. Blastomeres isolated from the animal half of sea urchin embryos and treated with lithium manifested a morphology resembling that of blastomeres deriving from the vegetal half of embryos. At the molecular level, lithium induced the appearance of molecular markers specific for the differentiation of vegetal structures, such as a gut specific enzyme and a RNA transcript encoding a skeletal spicule protein of larvae^24^. Furthermore, an increase was found in the expression level of the vegetal plate marker Endo16, when whole *Strongylocentrotus purpuratus* sea urchin embryos were treated with different concentrations of lithium. The increased expression level of this specific cell lineage marker paralleled the increase in the number of endodermal cells as a result of vegetalization^56^. Berridge and colleagues^57^ first suggested that lithium affects the phosphoinositide (PI) metabolism, i.e., the pathway generating the second messengers InsP_3_ and diacylglycerol (DAG) which regulate Ca^2+^ signaling and protein kinase C (PKC) activation, respectively^58^. The InsP_3_-PKC signaling pathway has been suggested to play a crucial role in vegetalizing the ectodermal cells in experiments in which a brief exposure of 16-cell stage sea urchin embryos to the PKC activator 12-O-tetradecanoyl phorbol- 13-acetate (TPA) enhanced their endoderm-mesoderm structures^59^. Significantly, then, the vegetalization process could also be induced by lithium. The phosphoinositide pathway is blocked by lithium at the level of the myo-inositol 1-phosphomonoesterase: the supply of inositol necessary for the resynthesis of phosphatidyl inositol (PI), and the subsequent synthesis of the immediate precursor of InsP_3_ and DAG, PIP_2_ are thus inhibited^60^. In line with the suggestion that the vegetalizing effect of lithium was due to the blockade of the phosphoinositide cycle, the inhibition of the development of embryos could be reversed by the injection of *myo*-inositol into *Xenopu*s and sea urchin embryos^27^,^30^,^61^. Considering that the PI cycle becomes activated immediately after fertilization in sea urchin eggs, the conclusion that the role for lithium in perturbing the PI metabolism and thus the mechanism of cell determination and differentiation is mediated by its effect on the PI cycle thus appears plausible.

Recent work has confirmed that the interplay of a Ca^2+^- PKC signaling pathway with actin cytoskeleton is essential for a variety of cell specification events in the early sea urchin embryos^62^. Studies on GSK-3, which phosphorylates β-catenin, a downstream effector of the Wnt signaling pathway that regulates fundamental aspects of development, including cell fate specification, and increases the level of DAG and PKC activity^63^, have suggested that lithium exert its vegetalizing effect by inhibiting the activity of the kinase^64^,^65^.

Our results expand previous investigations on the negative effects of lithium on the embryogenesis of sea urchin embryos. Sea urchins have been widely used as sensitive indicators of biochemical, morphological and physiological changes linked to environmental stressors, such as pesticides, heavy metals, ionizing radiations, ocean warming and acidification, metal nanoparticles and natural toxins^42^,^45^,^66^,^67^,^68^,^69^,^70^,^71^,^72^,^73^,^74^,^75^. Our results have shown that lithium is able to induce malformations in sea *P. lividus* urchin embryos, exerting a strong effect if the treatment is made before fertilization at concentrations much lower than those applied after fertilization. This indicates that lithium affects the early stages of the fertilization processes that determine if the embryo development will proceed normally^76^,^77^.

Another interesting finding made in the present work was the strong effect of the pre-incubation of unfertilized eggs with 80 mM lithium on sea urchin embryogenesis. The pretreatment blocked the embryonic development at the blastula stage with the subsequent death of the embryos. The effect was reversible. After all the washing times tested after fertilization the embryos were able to develop until the pluteus stage, even if some of them resulted malformed.

Our study has also provided new information on genes as molecular targets of lithium. Interestingly, we demonstrated that LiCl, at the molecular level affected different classes of genes at specifically developmental stages. These results suggest that LiCl exerts its negative effect on physiological processes by affecting genes that play a key role in a broad range of functional responses, such as stress, development, differentiation, skeletogenesis and detoxification processes (Fig. 7). Our results have shown that at the early blastula stage (21 hpf) lithium preferentially affected the expression levels of two stress genes, *HIF1A* and *p53,* as well as of that of *δ-2-catenin* which is involved in development and differentiation processes. In previous work the effects of diatom-derived polyunsaturated aldehydes (PUAs) had been tested on sea urchin embryos, and *p53*, *HIF1A* and *δ-2-catenin* have been shown by interactomic analysis to represent HUB genes^74^. HUB genes are viewed as important nodes in a network in which these three genes interact with many other genes. For instance, the p53 gene has a key role in regulating the cell cycle and functions as a tumor suppressor. It allows the repair or deletion of cells exposed to agents that cause DNA damage, like hypoxia, UVR, ROS or mutagens in the multicellular organism^78^,^79^,^80^,^81^. In sea urchins exposed to ultraviolet radiation the p53 gene product is down-regulation, leading to apoptosis^82^. HIF1A, a heterodimeric transcription factor, regulates cellular energy metabolism and angiogenesis in response to hypoxia^83^. In sea urchin the initial activation of aboral (situated at the opposite extremity from the mouth) genes depends directly on the redox sensitive transcription factor HIF1A^84^. The δ-2-catenin gene could also be a target of the toxic effect of lithium: its product is normally expressed in the brain and the catenin-presenilin interaction has implications for cadherin function and regulation of cell-to-cell adhesion^85^,^86^.

Other genes could also be lithium targets. The *p16* gene encodes for a small acidic protein involved in the formation of the biomineralized skeleton of sea urchin embryos and adults^41^. Recent studies have shown that this gene is also targeted by manganese and cadmium^87^ and by the diatom-derived hydroxyacid 5-HEPE^45^, confirming their important roles in skeletogenic processes. The finding that additional stress genes i.e., the *GS* gene which is responsible for the regulation of the glutamine synthetase activity in the metabolism of nitrogen are targeted by lithium supports the involvement of these genes in the stress response in sea urchin embryos. Marrone *et al.*^88^ have reported that at the prism stage (24 hpf) of sea urchin *P. lividus* embryos the treatment with the diatom-derived PUA decadienal up-regulated the *GS* gene. These findings were in line with studies in plants showing that salt stress affected glutamine synthetase activity and mRNA accumulation in potato plants in an organ-dependent manner^89^,^90^,^91^. The *cyt b (cytochrome b)* gene has also been suggested to intervene in the stress response in a single report in the literature which described its involvement in the response to the decadienal and 5-HEPE^45^,^88^. At prism stage (24 hpf) the expression of gene *sox9*, which is involved in left-right asymmetry processes during development and differentiation^92^, is affected by the treatment of sea urchin embryos with 15-HEPE^45^ and is also targeted by lithium. Finally, at the pluteus stage *hat* gene which is transiently expressed during the blastula stage as an early embryonic messengers^93^ is up-regulated. Its expression level has been found by us to increase in embryos treated with the three PUAs decadienal, heptadienal and octadienal^42^ and with the 5-HEPE^45^. In this contribution we have demonstrated how changes in gene expression levels may be considered early indicators of stressful conditions in the marine environment. As observed in most adaptive responses, the control of gene expression is tightly regulated in a fast response mode. This enables the cell to change the transcriptional pattern within minutes in the presence of stress and to return to the basal state after the stress is removed^94^. Our data suggest that LiCl is able to switch-on target genes at certain concentrations by a highly sophisticated mechanism. To be more detailed, we have probably detected effects on different genes at specifically developmental stages, as LiCl did not act directly on its target genes, but through the action of other genes belonging to the same functional pathway. Taking this into account, future efforts will focus on the analysis of the entire transcriptome and/or proteome to clarify the factors, e.g., mRNAs and/ or proteins, can be modulated by LiCl at specific developmental stages.

Furthermore, the aim of the present work was to contribute to the understanding of the morphological and molecular changes underlying the toxic effects of lithium on sea urchin embryogenesis. Lithium is widely used in patients with mood instability, but its mechanism of action still has obscure facets. The suggestion that its pharmacological effect involves the depletion of free myo-inositol appears plausible for the nervous tissue effects. In central neurons the activity of InsP_3_ and the Ca^2+^ release triggered by it are terminated by the dephosphorylation of the inositol monophosphate to inactive inositol. Since blood inositol is not available because of the blood-brain barrier, central neurons cannot replenish their plasma membrane with PIP_2_. This will prevent the formation of InsP_3_ and thus alter Ca^2+^ signals, somehow leading to the mood instability phenotype^60^. In line with this it should be mentioned that at fertilization of all animal species including humans, a sperm-induced signal transduction leads to the hydrolysis of PIP2 and to the formation of InsP_3_, which is suggested to be responsible for the release of Ca^2+^ in the fertilized eggs. A normal pattern and amount of Ca^2+^ release is a prerequisite necessary for the successful activation of the egg^95^,^96^. Lithium could cross the placental barrier of pregnant women treated with it for bipolar disorders, and could be associated with the increased risk of neonatal malformations^97^.

An interesting and novel aspect of our work is that the lithium action on sea urchin embryo development is evident even after a short exposure (10 minutes) of unfertilized sea urchin eggs to it. This indicates that lithium exerts its effect on a process during which an increase in PIP_2_ takes place following fertilization^98^,^99^. Our previous work in starfish eggs^100^ and sea urchin (unpublished results) had shown that the PIP_2_ increase levels lasting for several minutes after sperm addition correlated with the dynamic changes of actin cytoskeleton at the egg surface: The latter are necessary for the structural reorganization of the cortex of the fertilized eggs that determine the incorporation of the sperm and direct the mitotic division of the zygote^101^. Thus, sea urchin eggs will be a useful model to clarify if the toxic effect action of lithium on sea urchin development is mediated by PIP_2_ depletion at the time of fertilization.

## Additional Information

**How to cite this article**: Ruocco, N. *et al.* New insights into negative effects of lithium on sea urchin *Paracentrotus lividus* embryos. *Sci. Rep.* **6**, 32157; doi: 10.1038/srep32157 (2016).

## Supplementary Material

Supplementary Information
