# Supplementary material for: New insights into negative effects of lithium on sea urchin Paracentrotus lividus embryos
Source: Sci Rep. 2016 Aug 26;6:32157. doi: 10.1038/srep32157 (PMC4999890; doi:10.1038/srep32157)

**Manuscript ID: SREP-16-20019**

**Title: New insights into negative effects of lithium on sea urchin *Paracentrotus lividus* embryos**

**Authors:** Nadia Ruocco, Maria Costantini, Luigia Santella

**S1 Table.** Accession number, primer sequences and length of PCR amplified fragments are reported for the genes analyzed.

* Sequences were retrieved using www.spbase.org designing primers with the *S. purpuratus* sequences

** For each gene same primers reported in the reference have been used

*** Sequences were obtained from http://www.ncbi.nlm.nih.gov/Taxonomy/Browser/wwwtax.cgi designing primers with *P. lividus* sequences

**Supplementary Figure S1.** Percentage of embryos treated with LiCl 80 mM, washed at different times of development and observed at 1wpf


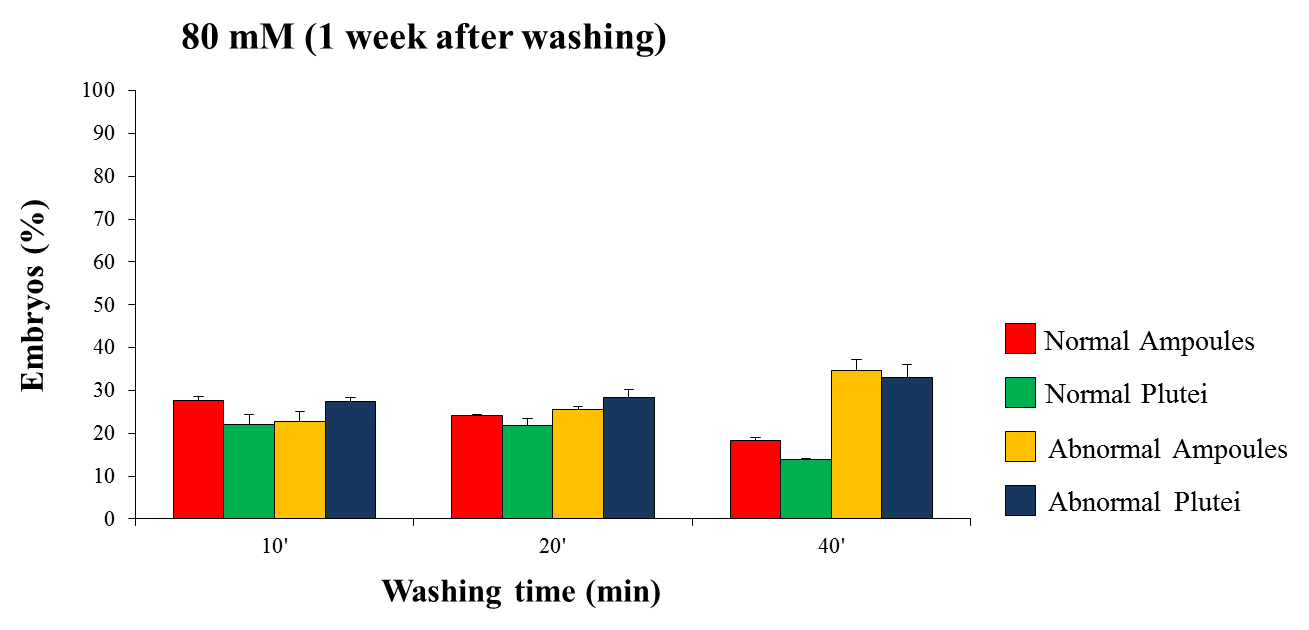


**Supplementary Figure S2.** Genes analyzed by Real Time qPCR, belonging to different functional classes: stress-related genes, genes involved in development and differentiation processes, genes involved in detoxification and skeletogenesis processes.


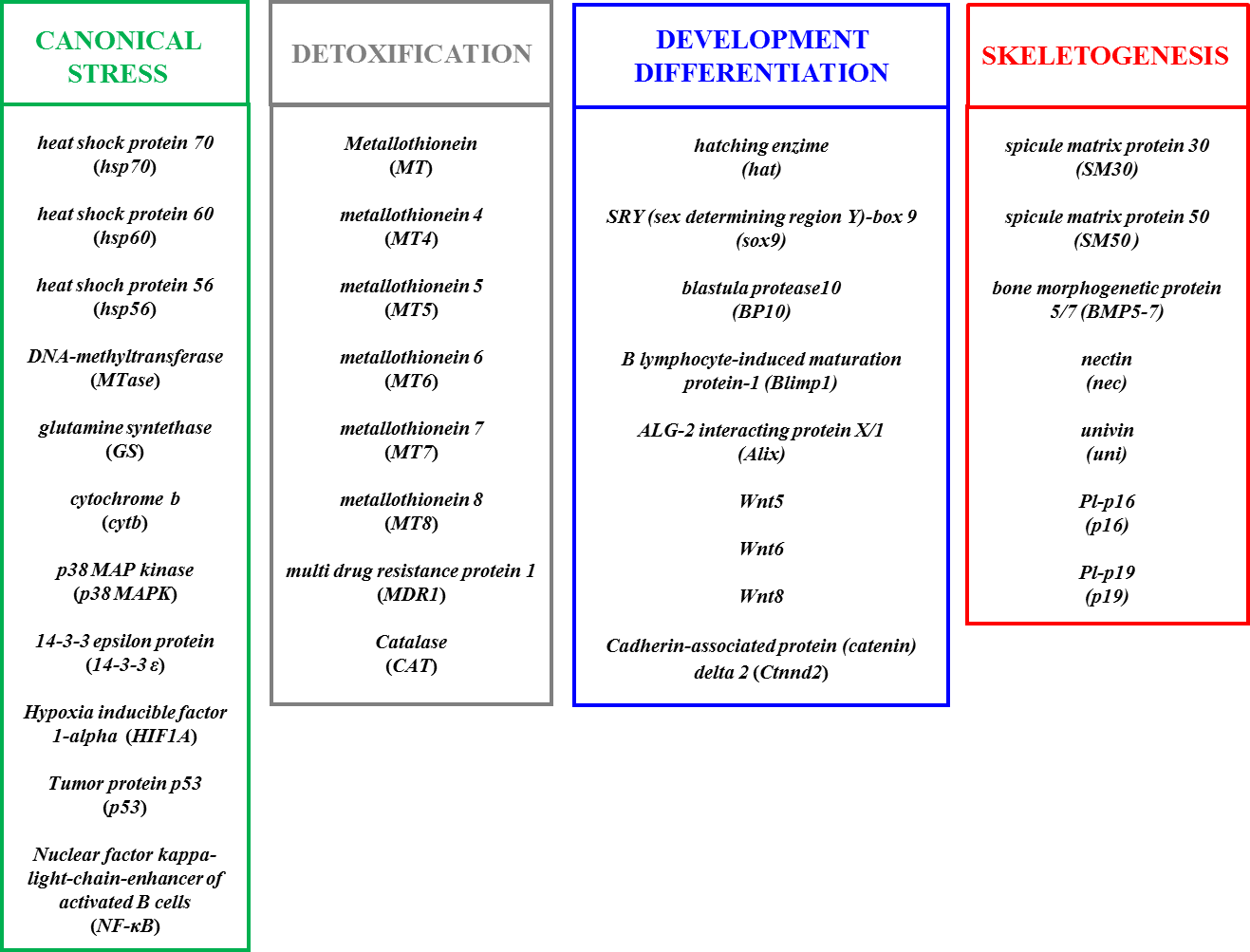

Supplement: Supplementary Information [file srep32157-s1.doc]
